# Appropriate Frequency and Interval of Neck Ultrasonography Surveillance during the First 10 Years after Total Thyroidectomy in Patients with Papillary Thyroid Carcinoma

**DOI:** 10.3389/fendo.2018.00079

**Published:** 2018-03-12

**Authors:** Yoo Jin Lee, Dong Wook Kim, Gi Won Shin, Young Jin Heo, Jin Wook Baek, Young Jun Cho, Young Mi Park, Ha Kyoung Park, Tae Kwun Ha, Do Hun Kim, Soo Jin Jung, Ji Sun Park, Ki Jung Ahn, Hye Jin Baek

**Affiliations:** ^1^Department of Radiology, Busan Paik Hospital, Inje University College of Medicine, Busan, South Korea; ^2^Department of General Surgery, Busan Paik Hospital, Inje University College of Medicine, Busan, South Korea; ^3^Department of Otorhinolaryngology-Head and Neck Surgery, Busan Paik Hospital, Inje University College of Medicine, Busan, South Korea; ^4^Department of Pathology, Busan Paik Hospital, Inje University College of Medicine, Busan, South Korea; ^5^Department of Nuclear Medicine, Busan Paik Hospital, Inje University College of Medicine, Busan, South Korea; ^6^Department of Radiation Oncology, Busan Paik Hospital, Inje University College of Medicine, Busan, South Korea; ^7^Department of Radiology, Gyeongsang National University School of Medicine, Gyeongsang National University Changwon Hospital, Changwon, South Korea

**Keywords:** papillary thyroid carcinoma, total thyroidectomy, ultrasonography, surveillance, recurrence

## Abstract

**Background:**

No previous study has employed the frequency and interval of follow-up ultrasonography (US) during the first 10 years after total thyroidectomy in patients with papillary thyroid carcinoma (PTC). The aim of this study was to determine the appropriate frequency and interval of follow-up US during the first 10 years in patients who have undergone total thyroidectomy for PTC.

**Methods:**

Two hundred seventy-two patients underwent total thyroidectomy for PTC at our institution from January 2006 to December 2007. Nineteen patients were excluded because of lack of US follow-up data for the neck. Follow-up US was performed by one of two radiologists in all patients. Tumor recurrence/persistence was confirmed by histopathology.

**Results:**

The mean interval between surgery and the final follow-up US examination was 79.0 months, and the mean number of follow-up US sessions was 5.9 in the 253 evaluable patients. Eleven patients (4.3%) developed tumor recurrence/persistence, which was detected on follow-up US within 5 years after total thyroidectomy in all cases. T and N stages were independently associated with tumor recurrence/persistence. The interval between surgery and first suspicion of tumor recurrence/persistence on follow-up US was ≤12 months in six patients and 20, 35, 41, 53, and 60 months in the remaining five patients.

**Conclusion:**

For detection of tumor recurrence/persistence after total thyroidectomy in patients with PTC, one or two sessions of follow-up US during the first 2 years, depending on T and N stages and one session of follow-up US in every second year during the following 8 years may be appropriate.

## Introduction

Up to 20% of patients with differentiated thyroid carcinoma develop locoregional recurrence (1, 2), which is fatal in 8% of cases (3). Ultrasonography (US) has been reported to have higher sensitivity for detection of postoperative recurrences of thyroid cancer in the neck than serum thyroglobulin or diagnostic whole-body scanning (4–7). Further, neck US is recommended as the first-line follow-up method in patients with differentiated thyroid cancer (4, 7). The recently published American Thyroid Association guidelines emphasize the role of neck US in follow-up of patients with papillary thyroid carcinoma (PTC) (8). In the current guidelines, it is recommended that these patients undergo neck US at least annually as part of routine follow-up, starting as early as 6 months after surgery, for detection of tumor recurrence or persistence (4, 8–10). However, the evidence for this short US follow-up interval is weak, and overuse of follow-up US examinations has economic implications.

In a recent multicenter study of follow-up US after total thyroidectomy for the treatment of PTC, the majority (66.1%) of cases of recurrence/persistence were detected within the first 2 years after total thyroidectomy (11). The authors of that study suggested that the optimal interval between thyroid surgery and the first US follow-up examination was 1–2 years and that one or two US surveillance examinations during the first five postoperative years may be appropriate (11). However, to the best of our knowledge, there have been no reports on the appropriate frequency and interval of US follow-up during the first 10 years after total thyroidectomy in patients with PTC. The aim of this study was to determine the appropriate frequency and interval of follow-up US during the first 10 postoperative years in patients who have undergone total thyroidectomy for PTC.

## Patients and Methods

### Patients

Two hundred seventy-two patients [252 women, 20 men; mean age 53.9 ± 12.1 (range 20–87) years] underwent total thyroidectomy for PTC at our hospital from January 2006 to December 2007. All patients routinely underwent postoperative neck US for detection of tumor recurrence/persistence or nodal metastasis. The patients who had undergone total thyroidectomy in 2007 had been included in a previous multicenter study that assessed postoperative tumor recurrence/persistence during 5 years of follow-up (11), which was extended to more than 10 years in this study. Nineteen of the 272 patients did not undergo postoperative follow-up US, so were excluded from the study, leaving data on 253 patients [234 women, 19 men; mean age 53.8 ± 12.0 (range 20–87) years] available for analysis. The institutional review board at our hospital approved the protocol used in this study (IRB: 17-0211). The need for informed consent was waived because of the retrospective nature.

### Total Thyroidectomy and Histopathology

In all cases, total thyroidectomy was performed by one of two surgeons with different levels of experience performing thyroid surgery (27 and 3 years). At our hospital, the extent of thyroid surgery is determined by multiple factors, including patient age, family history, tumor size, extrathyroidal tumor extension, nodal metastasis, and patient agreement. Total thyroidectomy was performed for patients with PTC and suspected perithyroidal tumor invasion or suspicious nodal metastasis on preoperative imaging, i.e., US or computed tomography. On histopathological analysis, the location and size of the primary PTC, perithyroidal extension, nodal metastasis, and multifocality were investigated. T and N stages were investigated on the basis of the eighth edition of the American Joint Committee on Cancer staging system (12).

### Follow-up US

Two experienced radiologists (undertaking more than 2,000 thyroid and neck US procedures per year) performed the follow-up US examinations of the neck using a high-resolution ultrasound instrument (HDI 5000 or iU 22; Philips Medical Systems, Bothell, WA, USA) equipped with a 5–15-MHz linear probe. At our hospital, follow-up neck US is routinely performed at 1- or 2-year intervals in patients who have undergone surgery for PTC. Features investigated on follow-up US examinations include the presence of a newly developed mass in the neck area, the presence of a suspicious lymph node in the neck, and interval change of the previous mass or lymph node in the neck. Known US features of nodal metastasis from PTC include diffusely increased echogenicity, intranodal microcalcifications, and an intranodal cystic component (13, 14).

### Tumor Recurrence/Persistence

In October 2017, single radiologist (DWK) retrospectively investigated US findings and histopathological results using a picture archiving and communication system and electronic medical records, respectively. Tumor recurrence/persistence was classified as non-nodal or nodal: non-nodal tumor recurrence/persistence was defined as the presence of a newly developed PTC in the postoperative thyroid bed, or perithyroidal neck area on histopathologic examination and nodal tumor recurrence/persistence was defined as nodal metastasis from PTC. Tumor recurrence/persistence was determined using US-guided fine-needle aspiration, core needle biopsy, or surgery for a suspicious neck mass or lymph node detected on follow-up US after total thyroidectomy.

### Statistical Analysis

The data were assessed for normality using the Kolmogorov–Smirnov test. Patient age, primary tumor size, number of follow-up sessions, and interval of postoperative US examinations are expressed as the mean ± SD. Differences in clinical and radiologic features between patients who did and did not develop tumor recurrence/persistence were evaluated using the independent *t*-test and Pearson’s χ^2^ test; for small cell values, Fisher’s exact test was used. Multivariate logistic regression analysis was used to determine the predictive power of individual clinical and radiologic features. The diagnostic performance of each potential predictor of tumor recurrence/persistence was evaluated by receiver-operating characteristic curve analysis. A cutoff value for each predictor was determined by maximizing the sum of sensitivity and specificity. The area under the receiver-operating characteristic curve was computed using the logistic regression model output. The statistical analyses were performed using SPSS version 24.0 (IBM Corp., Armonk, NY, USA) and MedCalc version 14.10 (MedCalc Software, Ostend, Belgium). A *p* value <0.05 was considered to be statistically significant.

## Results

The mean size of the primary PTC in the 253 patients was 12.3 ± 7.8 (range 2.0–45.0) mm. The primary PTC was located in the right lobe in 119 patients, the left lobe in 129, and the isthmus in 5. The T stage was T1a in 107 patients, T1b in 107, T2 in 31, T3a in 3, T3b in 5, T4a in 0, and T4b in 0. The N stage was N0 in 158 patients, N1a in 72, and N1b in 23. Multifocality and bilaterality were observed in 91 and 78 patients, respectively. The mean interval between total thyroidectomy and the last follow-up US examination was 79.0 ± 39.2 (range 5–137) months, and the mean number of follow-up US sessions was 5.9 ± 2.8 (range 1–14).

**Figure 1:**
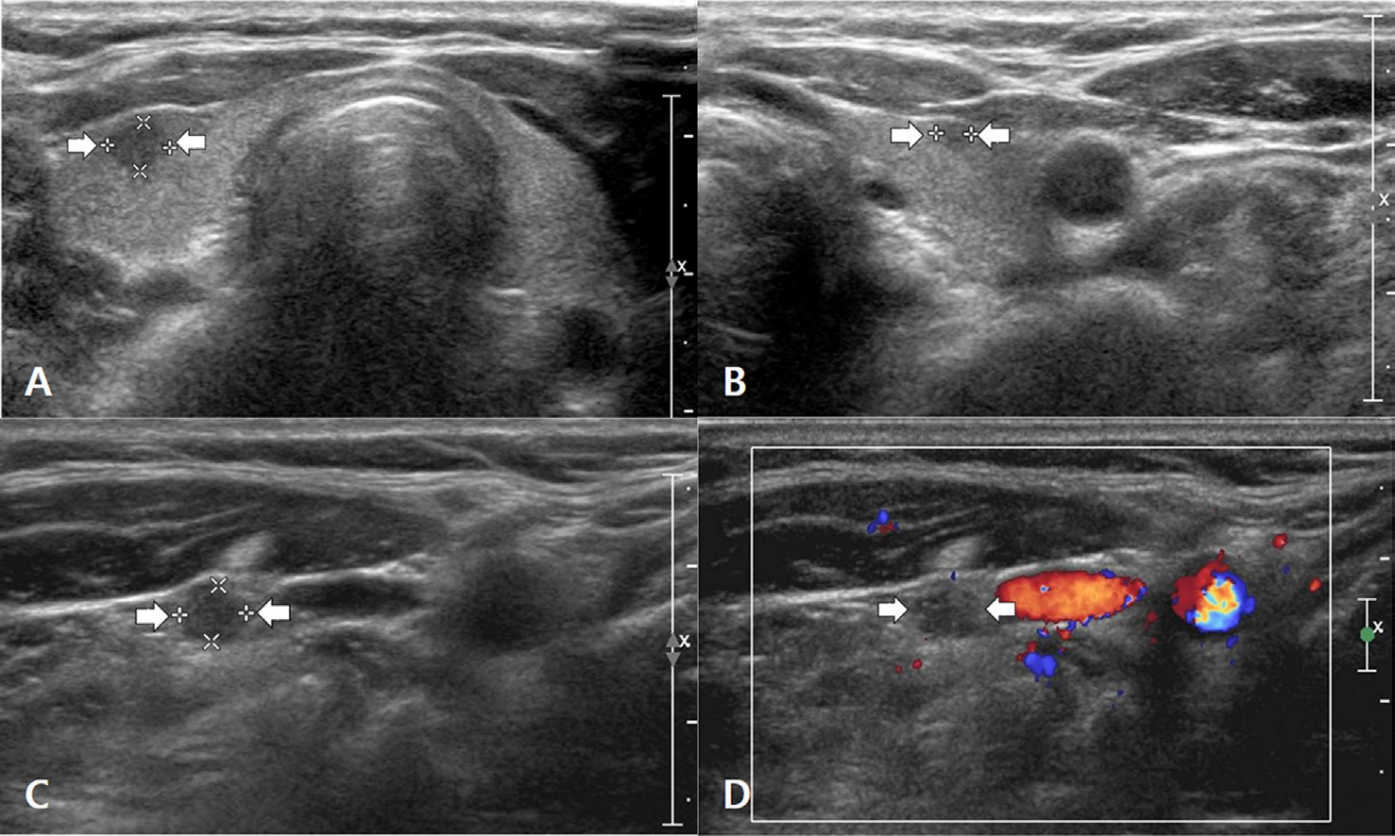
A 55- to 60-year-old woman with nodal tumor recurrence/persistence of papillary thyroid carcinoma (PTC). Before thyroid surgery, transverse gray-scale sonograms showed a primary PTC in the right lobe **(A)** (arrows, 3.6 mm × 4.5 mm × 4.7 mm) and a secondary PTC in the left lobe **(B)** (arrows, 2.0 mm × 2.4 mm × 2.7 mm). However, no suspicious lymph node was found on preoperative ultrasonography (US). On histopathologic examination after total thyroidectomy and central neck dissection, bilateral PTCs in both lobes and a single nodal metastasis in the right central neck were confirmed. On the 35-month follow-up US examination after total thyroidectomy, a transverse gray-scale sonogram **(C)** showed a suspicious lymph node with a round shape and diffusely increased echogenicity in the right mid-neck (arrows, 3.6 mm × 4.3 mm × 4.5 mm). On the transverse color Doppler sonogram **(D)**, the suspicious lymph node showed scant vascularity. After US-guided fine-needle aspiration for this node, nodal metastasis of PTC was diagnosed on cytology. After consecutive nodal dissection in the right neck, several metastatic nodes were confirmed on histopathologic analysis, and the patient underwent radioactive iodine ablation for the first time. There was no suspicion of tumor recurrence/persistence on subsequent follow-up US examinations (at intervals of 44, 56, 69, 82, 94, and 107 months).

Eleven (4.3%) of the 253 patients had tumor recurrence/persistence, which was nodal in 10 (Figure 1) and non-nodal in one. Tumor recurrence/persistence was confirmed by surgical excision after US-guided fine-needle aspiration. The mean largest diameter of the primary PTC and the recurrent/persistent lesion as measured on US was 16.1 (range 5.0–30.0) mm and 9.3 (range 4.9–15.4) mm, respectively. The majority (7/11, 63.6%) of recurrent/persistent lesions were subcentimetric in the largest diameter.

The clinical, US, and histopathologic findings in patients with and without tumor recurrence/persistence are compared in Table 1. There was a significant difference in T and N stages (*P* < 0.0001) and number of follow-up US sessions (*P* = 0.001) between the two groups, but not in patient age or sex, size, or location of the primary PTC, multifocality, or interval between surgery and the final follow-up US (*P* > 0.05).

**Table 1:** Comparison of clinical, ultrasonographic, and histopathologic results in 253 patients who underwent total thyroidectomy for PTC.

Items	TRP (−) (*n* = 242)	TRP (+) (*n* = 11)	*p* Value
Age (years, mean ± SD)	54.1 ± 11.8	47.2 ± 13.3	0.122
Sex (women:men)	223:19	11:0	1.000
Size of primary PTC (mm, mean ± SD)	12.2 ± 7.8	16.1 ± 7.7	0.104
Location of primary PTC			0.077
Right	112 (46.3)	7 (63.6)	
Left	126 (52.1)	3 (27.3)	
Isthmus	4 (1.7)	1 (9.1)	
T stage			<0.0001
T1a	104 (43.0)	3 (27.3)	
T1b	106 (43.8)	1 (9.1)	
T2	27 (11.2)	4 (36.4)	
T3a	1 (0.4)	2 (18.2)	
T3b	4 (1.7)	1 (9.1)	
T4a	0 (0)	0 (0)	
T4b	0 (0)	0 (0)	
N stage			<0.0001
N0	157 (64.9)	1 (9.1)	
N1a	66 (27.3)	6 (54.5)	
N1b	19 (7.9)	4 (36.4)	
Multifocality			0.264
Absence	157 (64.9)	5 (45.5)	
Presence	85 (35.2)	6 (54.5)	
Number of follow-up US session (mean ± SD)	5.8 ± 2.7	8.5 ± 2.4	0.001
Interval to the last follow-up US after total thyroidectomy (mean ± SD)	78.0 ± 39.2	99.8 ± 34.1	0.08

*Data are number of nodules, with percentage in parentheses*.

*NA, not applicable; TRP, tumor recurrence/persistence; PTC, papillary thyroid carcinoma; US, ultrasonography*.

The results for follow-up US and tumor recurrence/persistence are shown in Table 2. There was variation in the number of follow-up US sessions and in the interval between surgery and the last US follow-up examination. The interval between total thyroidectomy and the last follow-up examination was 120 months or more in 64 (25.3%) of the 253 patients. In the 11 patients with tumor recurrence/persistence, the mean interval between total thyroidectomy and the first detection of tumor recurrence/persistence on follow-up US was 23.5 ± 20.2 (range 6–60) months. In six (54.5%) of these patients, tumor recurrence/persistence was detected on follow-up US during the first postoperative year [mean 8.2 (range 6–11) months]; in the remaining five patients, the intervals until first detection of tumor recurrence/persistence on follow-up US were 20, 35, 41, 53, and 60 months.

**Table 2:** Follow-up US and tumor recurrence/persistence in 253 patients who underwent total thyroidectomy for papillary thyroid carcinoma.

Number of follow-up US sessions	Interval of the last follow-up US (month: mean ± SD, range)	Tumor recurrence/persistence (*n*)
1 (*n* = 18)	16.9 ± 14.7 (5–55)	0
2 (*n* = 22)	23.4 ± 6.8 (10–37)	0
3 (*n* = 18)	34.8 ± 9.0 (16–54)	1
4 (*n* = 18)	45.9 ± 12.4 (27–71)	0
5 (*n* = 34)	66.0 ± 15.8 (36–122)	0
6 (*n* = 24)	91.8 ± 23.9 (61–126)	0
7 (*n* = 39)	99.3 ± 17.7 (64–128)	2
8 (*n* = 42)	114.0 ± 11.6 (91–137)	1
9 (*n* = 17)	120.1 ± 8.5 (101–133)	4
10 (*n* = 11)	120.6 ± 8.1 (106–133)	1
11 (*n* = 6)	122.5 ± 8.8 (108–134)	1
12 (*n* = 3)	118.7 ± 12.7 (104–127)	1
13 (*n* = 0)	0 (0)	0
14 (*n* = 1)	127 (127)	0

*US, ultrasonography*.

In multivariate logistic regression analysis, T and N stages were significantly and independently associated with tumor recurrence/persistence (Table 3). Similarly, in the receiver-operating characteristic curve analysis, T and N stages showed a statistically significant ability to predict tumor recurrence/persistence (Table 4).

**Table 3:** Multivariate logistic regression analysis of factors predicting tumor recurrence/persistence in 253 patients who underwent total thyroidectomy for PTC.

Items	Odds ratio^a^	*p* Value
Patient age	1.03 (0.96, 1.10)	0.459
Sex	NA	NA
Size of primary PTC	1.13 (0.96, 1.33)	0.137
Location of primary PTC	2.33 (0.54, 10.12)	0.261
T stage	4.24 (1.35, 13.33)	0.013
N stage	3.47 (1.15, 10.42)	0.027
Multifocality	1.45 (0.85, 1.45)	0.172
Interval of the last follow-up US	1.03 (0.97, 1.08)	0.357
Number of follow-up US session	1.45 (0.96, 3.28)	0.069

*^a^Numbers in parentheses are 95% confidence intervals*.

*PTC, papillary thyroid carcinoma; US, ultrasonography*.

**Table 4:** Diagnostic performance of N stage as the best independent predictor of tumor recurrence/persistence in 253 patients who underwent total thyroidectomy for papillary thyroid carcinoma.

Items	*A_z_*value^a^	Sensitivity(%)	Specificity(%)	PPV(%)	NPV(%)	Cutoff^b^	*p* Value
T stage	0.722 (0.662, 0.776)	63.6	86.8	17.9	98.1	>T1b	0.031
N stage	0.807 (0.753, 0.854)	90.9	64.9	10.5	99.4	>N0	<0.0001

*^a^A_z_ indicates the largest area under the receiver-operating characteristic curve*.

*^b^Cutoff value demonstrated no metastasis as 0, N1a as 1, and N1b as 2*.

*Numbers in parentheses are 95% confidence intervals*.

*PPV, positive predictive value; NPV, negative predictive value*.

## Discussion

Neck US is the current gold standard for postoperative surveillance to detect tumor recurrence/persistence in patients who have undergone total thyroidectomy for PTC (8, 9). To date, the role of neck US is particularly important in patients who have remnant thyroid tissue after total thyroidectomy for PTC (15). In the literature, less than 1–2% of low-risk patients and 8% of intermediate-risk patients were identified to have structural recurrence/persistence of PTC (8), which involved lymph nodes in the neck in the majority of cases (60–75%) (1). In this study, the rate of tumor recurrence/persistence was 4.3% (11/253), which is within the range reported previously (8).

Known risk factors for recurrence of PTC include a large primary tumor, extrathyroidal invasion, advanced T stage, and advanced N stage (11, 16, 17). In this study, the risk factor for tumor recurrence/persistence was advanced T and N stages. The reason for this difference is unclear, but it may relate to the low prevalence of a large primary tumor and the low rate of tumor recurrence/persistence in our study.

All our cases of tumor recurrence/persistence were detected on follow-up US within 5 years after total thyroidectomy, and the majority (72.7%, 8/11) were detected on follow-up US within the first 3 years after surgery. Our findings suggest that the frequency of follow-up US examinations for detection of tumor recurrence in the 1–10 years after total thyroidectomy should be selected according to the T and N stages. However, our study included a small number of patients with high T and N stage disease. Therefore, we recommend one or two follow-up US examinations during the first 2 years, depending on T and N stages and one follow-up US examination in every second year during the subsequent 8 years.

This study had several limitations. First, not all patients underwent a final follow-up US examination >10-year period. In 189 patients (74.7%), the period between total thyroidectomy and the final follow-up US was less than 10 years. Second, the frequency and interval of follow-up US were variable and irregular. Third, not all the patients who underwent total thyroidectomy during the study period were included in the analysis. Of the 272 eligible patients, 19 (7.0%) were excluded because of absence of postoperative follow-up US. Fourth, no data for serology, radioactive iodine ablation therapy, computed tomography, or whole-body scans with radioiodine could be included. Finally, interobserver variability between the two radiologists who performed the follow-up US examinations could not be assessed because of the low prevalence of tumor recurrence/persistence in the study.

In conclusion, this study demonstrated a low rate of tumor recurrence/persistence after total thyroidectomy for PTC, that only T and N stages had a significant association with tumor recurrence/persistence, and that the majority of patients with recurrence/persistence were detected on follow-up US within 3 years after total thyroidectomy. Thus, annual follow-up US examinations of the neck for detection of tumor recurrence/persistence in the first 10 years after total thyroidectomy may be excessive. Therefore, we recommend one or two sessions of follow-up US during the first 2 years after total thyroidectomy, depending on T and N stages and one session of follow-up US in every second year during the subsequent 8 years. A large-scale multicenter study is required to confirm our hypothesis.

## Ethics Statement

All study participants waived informed consents owing to the retrospective analysis, and the study design was approved by the appropriate ethics review boards.

## Author Contributions

Concept and design: DWK. Acquisition of data: YJL and DWK. Literature review and refinement of manuscript: all the authors. Analysis and interpretation of data: HJB and DWK. Manuscript writing: YJL and DWK. Review of final manuscript: DWK.

## Conflict of Interest Statement

The authors declare that the research was conducted in the absence of any commercial or financial relationships that could be construed as a potential conflict of interest.
